# Research on user acceptance intention of artificial intelligence generated content for museum cultural and creative products

**DOI:** 10.1371/journal.pone.0349450

**Published:** 2026-06-10

**Authors:** Zhang Dongjiao, Zhang Yunlong, Jiang Xu

**Affiliations:** Academy of Arts & Design, Tsinghua University, Haidian, Beijing, China; Guilin University of Technology, CHINA

## Abstract

Recent advancements in the field of artificial intelligence have driven the rapid adoption of artificial intelligence generated content in the cultural and creative industries, making it a core driver of innovation. However, empirical research on user acceptance of artificial intelligence generated content remains limited. To address this research gap, this study, based on a review of the literature, constructs a four-dimensional analytical framework comprising technical functionality, cultural and aesthetic value, perceived psychological value, and user acceptance. Using structural equation modeling, this study analyzed data from 323 users with experience using artificial intelligence generated content to reveal the underlying relationships among these variables. The results indicate that technical functional experience has a significant positive impact on both user acceptance and cultural aesthetic value; cultural aesthetic value directly enhances psychological perceived value and positively influences user acceptance through a mediating effect; and psychological perceived value has a direct positive effect on user acceptance. The findings of this study provide empirical evidence to help stakeholders in the cultural and creative sector optimize their strategies for applying AI-generated content.

## 1 Introduction

In an era where digital technology is reshaping cultural dissemination, artificial intelligence generated content (hereinafter referred to as AIGC) is emerging as a core driver of innovation in museum cultural and creative products. Through the intelligent transformation of artifact patterns and historical symbols via algorithms, AIGC has not only overcome efficiency bottlenecks in traditional cultural and creative design but has also propelled a paradigm shift in cultural heritage from “static display” to “dynamic experience” [1].

The reason museum cultural and creative products have garnered widespread attention lies in their core differences from ordinary cultural and creative products, which manifest across three dimensions [2]: (1) Cultural Core: Museum cultural and creative products rely on the unique cultural symbols of the museum’s collection, embodying the scarce cultural value of a specific historical context, whereas ordinary cultural and creative products draw from a broader range of cultural symbols and lack the unique attributes of a museum collection [3]; (2) Functional positioning: Museum cultural and creative products combine cultural dissemination, public education, and commercial attributes, requiring a balance between cultural authenticity and market demand. Ordinary cultural and creative products are primarily guided by commercial value and user needs, with cultural dissemination serving as a secondary function [4]; (3) Emotional Connection: Museum cultural and creative products evoke users’ historical memories and cultural identity through artifact symbols, resulting in deeper and more unique emotional connections. In contrast, the emotional value of ordinary cultural and creative products often stems from superficial associations such as stylistic preferences and practical needs [5]. This uniqueness dictates that the application of AIGC in museum cultural and creative products must balance technical efficiency with cultural depth. Scholars from multiple countries have focused on researching the distinctiveness of museum cultural and creative products. For instance, an empirical study by Bao, Suomiya et al. on museums in the UK, Japan, and China confirmed that the core driver of user acceptance for museum cultural and creative products is cultural authenticity rather than technological novelty, with its explanatory power significantly exceeding that of technological attributes [6]; Cannavale C and Claudio L, in their study of China, Italy, Russia, and the United States, also noted that the application of AIGC in museum cultural and creative products, if detached from the premise of accurately translating cultural symbols, would lead to a significant decline in users’ cultural identification [7]. From the perspective of global research trends, interdisciplinary research on AIGC and cultural and creative products has formed three core strands. At the technological level, international research focuses on how AIGC enhances the design efficiency and innovation of general cultural and creative products; however, such studies do not account for the unique constraints of cultural authenticity inherent in museum cultural and creative products, making them difficult to directly adapt to museum contexts [8]. At the level of cultural translation, based on Hofstede’s theory of cultural dimensions, the collectivism and high power distance characteristic of Eastern cultures lead users to place greater emphasis on the social recognition value and authentic transmission of cultural products, whereas Western users place greater emphasis on personal aesthetic experiences and technological innovation [9]. Cross-cultural studies have addressed the issue of symbolic translation in general cultural and creative products using AIGC, but have rarely examined the impact of precise translation of museum artifact symbols on user acceptance. This disparity results in heterogeneous mechanisms of synergy between technological efficiency and cultural depth across Eastern and Western contexts [10]. At the user acceptance level, Western research has explored the drivers of acceptance for AI-driven cultural and creative products through Technology Acceptance Models (TAM, UTAUT), confirming the moderating effects of cultural value orientations and perceived risk on acceptance [11]. However, these studies have not distinguished between the acceptance differences of general cultural and creative products and those of museum-based cultural and creative products; existing models have not incorporated core variables specific to museum-based cultural and creative products, such as “perceived cultural authenticity” and “historical emotional resonance.”Although research in the global cultural and creative products sector has accumulated certain achievements, there remain three key gaps in the exploration of museum cultural and creative products: First, existing research largely focuses on the technical efficiency or market performance of general cultural and creative products, neglecting the core demand for cultural authenticity in museum cultural and creative products, which makes it difficult to adapt theoretical models [12]; second, there is a lack of comparative analysis from a broad perspective of cultural and creative products, failing to highlight the particularities of museum cultural and creative products in terms of acceptance mechanisms; third, there are obvious geographical limitations, as most specialized studies focus on a single country or region, lacking an analysis of commonalities and differences from a global perspective [13]. These findings collectively indicate that museum cultural and creative products differ fundamentally from general cultural and creative products. Focusing on this field does not represent a limitation in the scope of research but rather a precise response to its unique characteristics; consequently, research conclusions in this area offer more targeted insights for AIGC practices in museum cultural and creative products [14].

The theoretical framework of this study is constructed through the integration and extension of three major classical theories. It proposes a three-dimensional practical pathway of “Technology Optimization–Cultural Empowerment–Psychological Drive,” providing a systematic analytical perspective for integrating the characteristics of AIGC technology with those of the cultural context, and for deciphering the internal mechanisms through which dual stimuli are transformed into acceptance behavior via psychological perception [15]. One of its core values lies in addressing a classic controversy in the field of cultural technology studies: the debate between “technological instrumentalism” and “cultural determinism” [16]. By integrating TAM, VAM, and S-O-R theories, this study demonstrates that the experience of technological functionality does not directly drive behavior but rather exerts an indirect effect through the transformation of cultural aesthetic values and the mediating role of psychological perception. Furthermore, the mediating effect of cultural aesthetic values is significantly stronger than the direct effect of technology. This indicates that technology and culture are not in an antagonistic relationship but rather a synergistic transmission relationship involving tools, values, and psychology, providing empirical support from the museum cultural and creative context to resolve this controversy. While existing research has attempted to apply these theories to the field of digital cultural products, no integrated framework has yet been established for the AIGC + museum cultural and creative context. In particular, there is a lack of in-depth exploration of the mediating role of cultural aesthetic values, which provides scope for theoretical expansion in this study [17].

## 2. Literature review and hypothesis formulation

### 2.1 Construction of the core theoretical framework and hypothesis formulation

#### 2.1.1 Technology acceptance models and their evolution.

The Technology Acceptance Model (TAM), proposed by Davis in 1989, serves as the core framework for analyzing user technology acceptance behavior.The core logic of this model lies in the fact that Perceived Usefulness (PU) and Perceived Ease of Use (PEU), as key antecedent variables, significantly influence users’ willingness to adopt and actual usage behavior through the mediating effect of attitude [18]. Due to its simplicity and universality, this model has been widely applied in adoption studies across fields such as artificial intelligence and digital technology, becoming a benchmark framework in the field of technology acceptance.

As research contexts have diversified, scholars have gradually recognized the inherent limitations of the Technology Acceptance Model (TAM) in cross-cultural and complex cultural settings: First, its core variables focus on the instrumental attributes of the technology itself, neglecting the shaping role of contextual variables such as cultural values and social norms on acceptance behavior;Second, the model fails to account for differences in the relative weights of perceived usefulness and perceived ease of use on acceptance intentions across cultural contexts, making it difficult to explain variations in technology acceptance among cross-cultural groups; third, in scenarios emphasizing emotional and value-based identification (such as cultural creativity), the model lacks consideration of non-utilitarian variables.To address these limitations, the academic community has developed an extended version of the TAM model by integrating contextual variables and validated it in cross-cultural and technology convergence scenarios: Zhang LY et al. incorporated perceived risk and experiential openness into an integrated TAM-TPB framework to compare differences in AIGC acceptance between Chinese and American users.The study found that social norms exerted a significant positive influence on users in both countries; however, Chinese users placed greater emphasis on perceived usefulness and risk avoidance, whereas American users focused more on perceived ease of use and experiential openness [19]. Xia TS et al. introduced subjective norms and collaboration needs variables within the context of China’s collectivist culture, validating the explanatory power of the extended Technology Acceptance Model (TAM) for collaborative learning acceptance behaviors on social media [20].These studies indicate that the Technology Acceptance Model requires the incorporation of cultural context variables to adapt to cross-cultural and cultural scenarios. However, existing extensions have not yet formed a complete logical chain—‘technology attributes — cultural cognition — acceptance behavior’—making it difficult to directly apply the model to the specific context of AIGC + museum cultural and creative products.

The core logic of the TAM theory emphasizes the direct influence of technological attributes on acceptance behavior. Given the characteristics of AIGC cultural and creative scenarios, the experience of technological functionality serves as a fundamental prerequisite for user acceptance of the product. Experiences such as the fluidity and accuracy of technological functions can lower the operational threshold for users and enhance their perceived sense of control, thereby directly prompting users to engage in acceptance behaviors such as purchasing and recommending. Based on this, the following hypotheses are proposed:

H1: The experience of technical functionality has a positive influence on users’ behavioral intentions.

#### 2.1.2 Value-based adoption model and its value-mediation logic.

The Value-Based Adoption Model (VAM), proposed by Kim et al. (2017) based on the Technology Acceptance Model (TAM), represents a core innovation by transcending the limitations of focusing solely on technological attributes. It positions perceived value as the central mediating variable, establishing a transmission pathway of “technological attributes -perceived value-adoption behavior” [21].Compared to TAM, VAM is more suitable for research in cultural contexts. Its core advantage lies in emphasizing the mediating roles of affective and social value—these two types of value align closely with cultural values and cultural identity, providing a key entry point for analyzing technology acceptance mechanisms within cultural contexts.

Existing research has applied the Value-Affect-Motivation (VAM) model to fields such as digital cultural products and smart services: Shi et al. analyzed the drivers of purchase intention for AI-generated creative products based on VAM, confirming the synergistic mediating effect of functional value and affective value [22]; Erwin et al. combined the VAM with perceived risk theory and found that perceived value plays a full mediating role in the willingness to pay among mobile reading users [8]. However, the application of the VAM in cross-cultural and AIGC cultural contexts still has significant shortcomings: First, there is a lack of analysis of the interactive mechanisms between cultural values and perceived technological values, failing to elucidate how cultural contexts shape the dimensional weights of perceived value;Second, in the context of cultural and creative products, existing research has failed to distinguish the hierarchical relationship between “technological functional value” and “cultural aesthetic value,” making it difficult to explain the impact of cultural symbol transformation on value perception; third, most existing studies focus solely on single-cultural contexts and have not verified their cross-cultural adaptability, thus failing to provide theoretical support for the cross-cultural dissemination of AIGC cultural and creative products.

The core of the VAM theory lies in the transmission logic of “technological attributes - value perception - acceptance behavior.” In the context of AIGC culture and creativity, the experience of technological functionality serves as the cornerstone for the formation of cultural aesthetic value, which in turn directly drives user acceptance behavior. On the one hand, the precision of cultural symbol analysis and the effectiveness of technological transformation directly influence the level of cultural aesthetic value; on the other hand, the enhancement of cultural aesthetic value strengthens users’ value identification, thereby promoting acceptance behavior.

#### 2.1.3 The connotation, theoretical basis, and mediating mechanisms of cultural aesthetic value and cultural value perception.

Cultural aesthetic value refers to users’ comprehensive evaluation of AIGC – generated museum cultural and creative products in terms of formal beauty, the expression of cultural symbols, and emotional experience; it serves as a key cognitive variable linking technological functionality and psychological perception. This study defines and deconstructs this concept from three perspectives: aesthetic theory, aesthetic psychology, and cultural consumption theory. First, based on modern aesthetics and the theory of disinterested aesthetics, cultural aesthetic value emphasizes the unity of formal beauty and cultural significance. It encompasses not only visual elements such as color schemes, composition, and symbolic harmony but also the historical memories and aesthetic experiences evoked by cultural relic symbols [23]. Contemporary aesthetic research indicates that the aesthetic value of digital cultural products is no longer confined to formal appearance but places greater emphasis on the accurate translation of cultural meaning and the generation of emotional resonance, providing direct grounds for defining the connotation of cultural aesthetic value. Second, drawing on psychological distance theory, the cultural aesthetics of museum cultural and creative products must maintain an appropriate distance between “cultural authenticity” and “modern aesthetic preferences”—neither detaching from the historical context of cultural relics nor failing to adapt to the aesthetic and reception habits of contemporary users [24]. A moderate psychological distance can enhance the intensity of the aesthetic experience and promote users’ identification with and acceptance of cultural products. Finally, based on cultural capital and cultural consumption theories, users’ recognition of cultural aesthetic value is essentially an identification with and internalization of cultural capital, cultural identity, and cultural significance [25].

As a core mediating variable, the perception of cultural value shares a common lineage with cultural aesthetic value and can be deconstructed into three dimensions: the accuracy of cultural symbol translation, the intensity of emotional resonance, and the degree of cultural identification. It represents the deepening and implementation of cultural aesthetic value at the psychological level of users. Existing research has confirmed its mediating role in contexts such as technology acceptance and cultural consumption; however, its mechanism of action in the AIGC + museum cultural and creative context remains to be clarified. Regarding its dimensional composition, scholars have refined it based on different contexts. Feng XZ et al. defined traditional cultural cognition as comprising two core dimensions: cultural symbol recognition and understanding of cultural connotations, demonstrating that it positively influences digital literacy through a chain-mediated effect via technology acceptance [26]; Ji Zhongyang from the perspective of intangible cultural heritage aesthetics, proposed that cultural value cognition should encompass two dimensions: aesthetic identification and cultural heritage identification [27]. While these studies provide references for defining dimensions, they have not yet formed a unified framework applicable to AIGC cultural and creative scenarios, nor have they considered the impact of technology-generated characteristics on the accuracy of cultural symbol translation; Regarding mediating effects, existing research has established the basic logic of “technological functionality – perception of cultural value – behavioral response.” Singh G et al. found that the perception of cultural appropriateness in service contexts mediates the relationship between service quality and user behavior [28], Pang C et al. confirmed that cultural identity mediates the relationship between family cultural capital and the intention to purchase cultural and creative products [29], while Zang Zhipeng et al. proposed that the immersive experience of digital cultural products influences user acceptance through the mediation of cultural identity [30]. However, these studies have not explicitly identified the chain-mediation pathways in AIGC scenarios, nor have they considered the moderating role of cultural context.

Given the characteristics of the AIGC cultural and creative context, the experience of technical functionality serves as the foundation for the formation of cultural aesthetic value and cultural value perception: the fluidity and accuracy of AIGC algorithms in precisely analyzing the features of cultural relics ensure the authenticity of cultural symbols in product design, which is a prerequisite for the formation of cultural aesthetic value and cultural value perception [31]; simultaneously, the innovative combination of algorithms drives the integration of traditional cultural elements with modern design styles, enhancing the uniqueness and appeal of cultural aesthetic value, thereby reinforcing users’ cultural value cognition [32]. The enhancement of cultural aesthetic value and cultural value cognition, in turn, strengthens users’ value identification—users form a positive attitude toward the product by perceiving the accuracy of cultural symbol translation and the intensity of emotional resonance, which subsequently triggers acceptance behaviors such as purchasing and recommending.

Based on the aforementioned theoretical and research foundations, cultural aesthetic value serves as the core external manifestation of cultural value perception. Regarding its relationship with technical functional experience and user behavioral intent, the following hypotheses can be proposed:

H2: Technical functional experience positively influences cultural aesthetic value.

H3: Cultural aesthetic value positively influences user behavioral intent.

#### 2.1.4 The stimulus-organism-response (S-O-R) model and its contextual transmission framework.

In 1974, Mehrabian and Russell proposed the Stimulus-Organism-Response (S-O-R) model, which is based on the core logic that external environmental stimuli activate an individual’s internal psychological state, ultimately triggering specific behavioral responses. This model transcends the analysis of linear relationships between single variables, emphasizes the transmission chain of “environment - psychology - behavior,” and provides a systematic analytical perspective for understanding user behavior in complex contexts.

In scenarios where digital technology and culture converge, the application of the S-O-R model has gradually increased but remains fragmented: Yin Jie et al. used the digital technological attributes of museums as the stimulus source, emotional arousal as the organism, and visitors’ online engagement behaviors as the response, revealing the S-O-R transmission mechanism in cultural contexts [33]; Wu Tao et al. on the other hand, treated the technical and content characteristics of video platforms as dual stimulus sources, perceived value as the organism, and subscription intent as the response, thereby validating the model’s applicability in digital service scenarios [34].

This study adopts the S-O-R model to construct an ideal framework integrating both technological and cultural dimensions: the technical functional characteristics and cultural contextual features of AIGC can be regarded as dual external stimuli; cultural aesthetic value and user psychological perception constitute the organism; and user acceptance willingness and usage behavior serve as the final response, thereby forming a multidimensional, multilevel transmission mechanism.

The O-R model emphasizes the transmission logic of“stimulus - organism - response.” In this study, cultural aesthetic value serves as the key link in the transformation from “stimulus”to“organism,” while user psychological perception corresponds to the core “organism” variable. Users internalize cultural aesthetic values to form subjective psychological states, which in turn drive behavioral choices.Enhancing cultural aesthetic value can stimulate positive psychological perceptions in users, such as a sense of cultural identity and aesthetic pleasure, and these positive perceptions can directly prompt acceptance behavior.

#### 2.1.5 The conceptual framework and theoretical research on psychological perception.

As the core “organism” variable in the S-O-R model, the theoretical framework of user psychological perception is not a simple superposition of multiple theories. Instead, it integrates trust theory, cultural identity theory, self-determination theory, and aesthetic pleasure theory into a coherent system centered on the “process of forming users’ psychological responses to AIGC - generated museum cultural and creative products.” Each theory has its own distinct role yet is interconnected, collectively explaining the formation mechanism of psychological perception:

From an integrative perspective, these four theories form a complete chain of “foundational premises—core essence—intrinsic motivation—emotional supplementation”: Trust theory provides the foundational premises for psychological perception—for AIGC – generated museum cultural and creative products, users’ trust in the accuracy of algorithmic translation of cultural symbols and the authenticity of cultural connotations is the primary condition for forming positive psychological perception; a lack of trust will directly inhibit subsequent psychological reactions [11]; Cultural Identity Theory constitutes the core of psychological perception—the core value of museum cultural and creative products lies in cultural transmission. Users develop a sense of belonging and identity with their own culture through perceiving the cultural connotations within the products, which is the key feature distinguishing psychological perception from ordinary product experiences;Self-determination theory provides the intrinsic motivation for psychological perception—AIGC ’s support for personalized design (such as allowing users to participate in the design process) satisfies users’ need for autonomy, while the sense of accomplishment derived from understanding cultural connotations and engaging in creative interactions fulfills their need for competence. The satisfaction of these two needs drives the continuous deepening of psychological perception [35];Aesthetic pleasure theory complements the emotional dimension of psychological perception—users derive aesthetic pleasure from the formal and cultural beauty of the product. This emotional experience, combined with trust, cultural identity, and the fulfillment of motivational needs, further reinforces positive psychological perception.

Empirical research by Zhang, Xinyi et al. also indicates that there is a significant interactive effect between aesthetic pleasure and cultural identity, and that both can indirectly enhance the level of psychological perception through the fulfillment of self-determination needs [36], providing empirical evidence for the coherence of this theoretical integration.The S-O-R model emphasizes the transmission logic of “stimulus–organism–response.” In the context of this study, cultural aesthetic value serves as a critical link in the transformation from “stimulus” to “organism,” where users’ psychological perceptions form subjective psychological states through the internalization of cultural aesthetic value. The enhancement of cultural aesthetic value can stimulate positive psychological perceptions such as cultural identity, aesthetic pleasure, and perceived trust. Based on this, the following hypothesis is proposed:

H4: Cultural aesthetic value positively influences users’ psychological perceptions.

H5: User psychological perceptions positively influence users’ behavioral intentions.

### 2.2 Mechanisms of AIGC technology acceptance in a cultural context

As the application of AIGC technology in the cultural sphere continues to expand, the role of cultural context in shaping its acceptance mechanisms has gradually become a focal point of research. Existing studies primarily revolve around two core questions—“How does cultural context influence AIGC acceptance?” and “Pathways for AIGC cross-cultural adaptation”—and while a preliminary research consensus has been established, numerous research gaps remain.

Regarding the mechanisms by which cultural background influences AIGC acceptance, existing research confirms that cultural dimensions significantly moderate the core drivers of technology acceptance: Wang S., based on a sample from 20 countries, found that power distance and individualism within national cultural dimensions are significantly correlated with individual acceptance of artificial intelligence—groups with lower power distance and stronger individualism exhibit higher acceptance of AIGC [37]; Strandt et al. further found that differences in cultural value orientations between Eastern and Western leaders lead to significant variations in the core drivers of AI acceptance—Eastern leaders place greater emphasis on the alignment between technology and organizational culture, while Western leaders prioritize technological efficiency [38]. These studies confirm the moderating role of cultural background in AIGC acceptance mechanisms, but none have focused on the specific context of cultural and creative products, nor have they considered the influence of core cultural variables such as cultural symbol transformation and cultural identity.

Regarding the cross-cultural adaptation pathways for AIGC, scholars have proposed preliminary solutions from a technical optimization perspective: Dong Z constructed a multilingual text corpus by integrating natural language processing and sentiment analysis technologies, enabling symbolic substitution and adaptive tone adjustments, which increased the positive sentiment feedback rate for cross-cultural AIGC content by 35% and reduced the cultural misunderstanding rate by 42% [39]; Filipovic et al. found that the promotional effect of AI elements on social media user engagement is positively moderated by globalist cultural characteristics, suggesting that AIGC content design should incorporate dynamic cultural adaptation modules [40]. However, existing research still has limitations, such as a lack of quantitative studies on differences in cross-cultural consumer emotional needs and unclear definitions of authenticity thresholds for cultural symbol translation. These factors make it difficult to directly guide the AIGC design of museum cultural and creative products.

### 2.3 Research on the integration of AIGC and museum cultural and creative products

Innovations in digital technology have driven the deep integration of AIGC with the design of museum cultural and creative products. Existing research primarily focuses on the application value, technical pathways, and existing issues of AIGC in cultural and creative design, providing a practical foundation for this study; however, significant shortcomings remain.

Regarding technical application pathways, scholars have proposed various optimization schemes: Wang T et al.enhanced the alignment between rattan cultural and creative designs and consumer emotional cognition by integrating Grey Relational Analysis (GRA) with the intuitive fuzzy VIKOR method [41]; Abouelela et al.utilized AI technology to integrate Arabic calligraphy into modern furniture design, thereby strengthening the products’ sense of cultural belonging [42]; Liu Z et al. proposed, through Importance-Performance Analysis (IPA), that AIGC -driven cultural and creative design requires optimization in both cultural expression and user experience to enhance consumer satisfaction [43]. However, existing research primarily focuses on individual technology application cases and lacks quantitative analysis of the functional experience dimensions of these technologies, particularly regarding their mechanisms of action in influencing user behavior and the transmission of cultural values.

Regarding existing issues, research generally identifies four major challenges: First, a mechanism for distributing benefits among platforms, copyright holders, and users has yet to be established in the digital copyright economy. The root cause lies primarily in the ambiguous definition of copyright ownership for AIGC-generated content; existing laws struggle to accommodate the characteristics of human-machine collaborative creation, and there is a lack of regulations specifically tailored to the context of museum cultural and creative products.Hu, L. et al. note that the controversy over defining the “autonomy of algorithms” versus the “degree of human intervention” in AIGC creation is the core reason why copyright attribution remains unclear [44], while Zhang, J., & Li, Y. et al. also confirmed through their research on museum cultural and creative products that legal gaps exist regarding the copyright protection of symbols derived from museum collections and the rights allocation for AIGC – generated content, directly hindering the establishment of a profit-sharing mechanism [45]. Secondly, the lack of quantitative research on differences in the emotional needs of cross-cultural consumers makes it difficult to effectively support the cross-cultural dissemination of AIGC cultural and creative products [46]. This is primarily due to the lack of standardized emotional measurement tools in multilingual and multicultural contexts, the failure to integrate interdisciplinary empirical paradigms from neuroscience and cultural psychology into AIGC product testing processes, and constraints on cross-cultural sample collection imposed by ethical review and data sovereignty regulations. Research by Sharma, K., et al. on AIGC cultural and creative products also confirms that the quantitative analysis of cross-cultural emotional needs has resulted in limited empirical research accumulation due to the absence of standardized tools [47]; third, the creative industries lack standards for human-machine collaboration, making it difficult to ensure the cultural authenticity of AIGC content [11,33–40,42–48]. The root of this problem lies in the Western – centric bias present in the training data of current AIGC models, the limited ability to decode cultural symbols due to insufficient semantic granularity, and the absence of a collaborative annotation and feedback loop mechanism involving experts from the museum sector.Wu, H., et al. argue that when applying AIGC to the design of museum cultural and creative products, the challenge lies in integrating tradition and modernity, requiring a design framework that automates the extraction of cultural elements, generates design concepts, and optimizes aesthetics [49]. Fourth, theoretical research suffers from issues such as a narrow technological perspective, a high absence of cultural dimensions, and insufficient coverage of empirical samples [50]. This challenge stems from disciplinary barriers: AIGC research, dominated by computer science, overlooks theoretical frameworks such as cultural hermeneutics and heritage communication studies. Cultural dimension modeling relies on the recognition of superficial symbols rather than deep value structures, and empirical studies are often concentrated on single institutions or short-term experiments, lacking mixed-method designs that include long-term tracking and cross-museum comparisons.Zhan, X. argues that current analyses of AIGC applications in the cultural industry primarily emphasize the innovation and efficiency gains brought by technological adoption, while also highlighting the challenges it faces [51].

### 2.4 Theoretical integration logic and expansion of the “culture–technology–value–reception” model

The application of classical technology acceptance theory in the field of cross-cultural artificial intelligence exhibits an evolutionary trend of “basic application — contextual extension — multi-theory integration,” yet current research still suffers from core deficiencies such as fragmented theoretical application and a lack of systematic integration. Based on this, this study constructs an integrated model that achieves systematic integration of multiple theories and elucidates its logic of extension and integration with existing frameworks.

In addition to the aforementioned cross-cultural extension of the TAM model, research on the cultural context optimization of the UTAUT model has also gradually deepened: Meiryani et al. revealed significant differences in AI acceptance between Eastern and Western leaders through their cross-cultural study based on UTAUT [52]. The application of the VAM model and the S-O-R model remains in its early stages but shows great potential.Although existing research has not yet directly applied the VAM model to cross-cultural AIGC acceptance studies, Lee et al. implicitly followed its core logic in their study on how cultural cognition influences digital literacy through technology acceptance [53]; while the S-O-R model has been validated in single-culture settings, it has not yet formed a cross-cultural integration framework.

To address these shortcomings, this study constructs an integrated “Culture-Technology-Value-Acceptance” model, treating the technological functional characteristics of AIGC and cultural environmental characteristics as dual external stimuli, cultural value perception and perceived technological value as the organism, and users’ willingness to accept AIGC and usage behavior as the final response.Simultaneously, this model integrates the core variables of the Technology Acceptance Model (TAM) with the value perception dimension of the Value Acceptance Model (VAM) to address the explanatory limitations of single-theory approaches. This integrated model achieves the expansion and innovation of existing theoretical frameworks through three pathways: a TAM model that overcomes the limitations of technocentrism; the incorporation of cultural value perception into the core mediating chain; and the construction of an extended pathway of “technological attributes — cultural perception — acceptance behavior”;The VAM model, which expands the value perception dimension, combines cultural value perception with perceived technological value and elucidates the influence of cultural context on the formation of value perception; it deepens the cultural adaptability of the S-O-R model, proposes an expanded framework of “dual stimuli — composite organism — cross-cultural response,” and analyzes the critical role of cultural factors in stimulus transmission and organism activation.

In summary, the three classic models—TAM, VAM, and S-O-R—all exhibit significant limitations in the AIGC + museum cultural and creative context. Furthermore, previous research has largely applied single models in a fragmented manner, failing to establish an integrated framework that accounts for technology, culture, and psychology. The CTVA integrated model proposed in this study, through the targeted optimization strategies outlined in the comparison table above, effectively addresses the shortcomings of single models—— addressing the core issues of TAM ’s lack of cultural mediation, VAM ’s failure to clarify the hierarchy of cultural values, and the fragmented application of S-O-R, while integrating the strengths of the three models. It constructs a complete theoretical pathway of “technological functional experience - cultural aesthetic value - psychological perceptual value - user acceptance,” clarifies the intrinsic relationships among variables, and precisely fills the theoretical gaps in existing research. For a detailed comparison of the models, see Table 1.

**Table 1:** Theoretical comparison table.

Classic Models	Research Focus	Core Limitations	Solutions Proposed by the CTVA Integrated Model
TAM (Technology Acceptance Model)	Focuses on the direct influence of technology perception on user acceptance, with a core emphasis on the functional aspects of technology	1. Lacks cultural mediating variables, making it unsuitable for the cultural attributes of museum cultural and creative products; 2. Ignores the influence of non-utilitarian values; 3. Struggles to explain the pathway from technological functionality to cultural acceptance	1. Introduce cultural and aesthetic value as a core mediator to bridge the gap between technological functionality and psychological perception; 2. Incorporate cultural context variables tailored to the museum cultural and creative product setting to move beyond a purely technology-oriented approach; 3. Construct a comprehensive pathway: “Technology - Culture — Psychology — Acceptance”
VAM (Value Acceptance Model)	Focuses on perceived value as the core mediator, examining the impact of value transmission on user behavior	1. Fails to define a hierarchical structure of cultural values in AIGC scenarios, making it impossible to distinguish between cultural-aesthetic and general perceived values; 2. Does not clarify the prior driving role of technological factors on value perception; 3. Lacks adaptability to cultural product scenarios	1. Clarify the hierarchy of cultural aesthetic values; 2. Treat the technological functional experience as a preceding variable to refine the “Technology — Value — Acceptance” transmission logic; 3. Optimize the adaptability of the value dimension by integrating the characteristics of AIGC-assisted design
S-O-R (Stimulus-Organism-Response Model)	Emphasizes the chain-like transmission of “environmental stimulus — psychological state — behavioral response,” focusing on the connection between context and psychology	1. Fragmented application in cultural product contexts, failing to clarify the dual role of technological and cultural stimuli; 2. Failure to define specific variables at the “organism” level (e.g., cultural aesthetics, psychological perception); 3. Lack of targeted design for stimulus-response pathways within cultural contexts	1. Integrate AIGC technological stimuli with museum cultural stimuli to clarify the synergistic effects of dual stimuli; 2. Define cultural aesthetic value and psychological perception value as core variables at the “organism” level; 3. Adapt to AIGC + cultural and creative scenarios to construct a targeted “dual stimuli — psychological mediation — reception behavior” pathway

### 2.5 Proposed theoretical model

**Fig 1:**
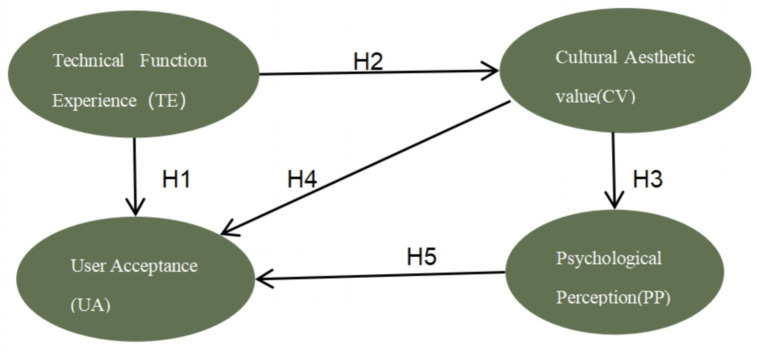
Conceptual model. The model illustrates the relationships among four key variables: Technical Function Experience (TE), Cultural Aesthetic Value (CV), Psychological Perception (PP), and User Acceptance (UA). Arrows indicate the direction of hypothesized causal relationships (H1 to H5). Specifically, TE is hypothesized to directly influence UA (H1) and CV (H2); CV is hypothesized to directly influence PP (H3) and UA (H4); and PP is hypothesized to directly influence UA (H5), potentially serving as a mediating variable within the framework.

Based on the above theory, this study proposes a comprehensive model (Fig 1) by fully considering the characteristics of museums and drawing on the techniques, methods, and findings of previous research. This model encompasses four dimensions: Technical Functional Experience (TE), Cultural Aesthetic Value (CV), Psychological Perception (PP), and User Acceptance (UA), and puts forward five related hypotheses to explore users’ satisfaction and purchase intent regarding AIGC-generated museum cultural and creative products.

## 3 Methods

### 3.1 Research design and methodology

This study clarifies the research questions through an extensive literature review, summarizes the research methods, explores the factors influencing users’ perceptions of the design of cultural and creative products, and proposes research hypotheses. User acceptance was collected via a questionnaire survey, with SPSS 26.0 and AMOS 24.0 serving as the core analytical tools. The specific analysis process is as follows: 1. Descriptive statistical analysis, used to present the demographic characteristics of the sample and the basic information of each construct;2. Reliability and validity testing: The questionnaire’s reliability and validity were verified using Cronbach’s alpha (reliability), exploratory factor analysis (EFA), and confirmatory factor analysis (CFA) (validity); 3. Correlation analysis: Pearson correlation analysis was used to examine the correlations among the constructs;4. Hypothesis testing: Structural Equation Modeling (SEM) is used to test the main effect hypotheses (H1–H5), and the Bootstrap method (5,000 repeated samples) is employed to test mediation effects, clarifying the transmission mechanisms among variables. For mediation effect testing, a bias-corrected 95% confidence interval is used; if the confidence interval does not include 0, the mediation effect is significant

### 3.2 Questionnaire survey

The questionnaire design for this study is based on the Technology Acceptance Model (TAM), the Value Acceptance Model (VAM), and the Stimulus – Organism – Response (S-O-R) models as its core theoretical framework. It integrates measurement items for each construct to ensure theoretical coherence and conceptual consistency in item selection. Centered on the hypothesized pathway of “Technology Functional Experience — Cultural Aesthetic Value — Psychological Perception — User Behavioral Intention,” the study ensures that all construct items align with their corresponding theoretical dimensions and are supported by established scales. The questionnaire employs a 5-point Likert scale (1 = Strongly Disagree, 5 = Strongly Agree) for measurement. Numerous statistical and methodological studies, such as those by Kusmaryono, Imam, et al. [54] and Gordon W. et al. [55], have confirmed the 5-point Likert scale’s suitability for SEM.

The questionnaire consists of two parts: Part One focuses on consumers’ perceptions of AIGC and their attitudes toward AI-generated designs, while also collecting demographic information from respondents; Part Two targets AI-generated museum cultural and creative products, employing a 5-point Likert scale to conduct an in-depth evaluation across two dimensions: importance and satisfaction. This scale performs well in balancing measurement accuracy and conciseness, and is therefore widely used across multiple research fields.During the survey, researchers provided respondents with a detailed explanation of the AI design proposals and creative process, guiding them to objectively evaluate their experience using a rating scale ranging from 1 (strongly disagree) to 5 (strongly agree).

After finalizing the design, this study employed random sampling to conduct a questionnaire survey and systematically collect consumer satisfaction data. To ensure the rigor of the measurement scale, the following scale development and pre-testing procedures were implemented: (1) Item Generation: Initial measurement items were drafted based on an extensive literature review, followed by an invitation to three experts to conduct a content validity review.The experts assessed the relevance, clarity, and comprehensiveness of each item in relation to its corresponding latent variable, provided revision suggestions, and ultimately finalized 25 initial items. (2) Pilot testing: A pilot survey was conducted among 50 eligible respondents (aged 18–45, with experience consuming museum cultural and creative products and familiarity with AIGC).Item analysis of the collected data: The critical ratio (CR) method was used to test item discrimination; items with CR < 3.0 (p > 0.05) were excluded. The correlation coefficients between each item and the total dimension score were calculated, and items with a correlation coefficient < 0.5 were removed. Following the pilot test, five items with poor discrimination and low factor loadings were excluded.(3) Cross-validation: The final scale was cross-validated using data from the main survey (n = 323). Confirmatory factor analysis (CFA) results showed that the factor loadings for all items were greater than 0.6 (see Table 5), and the average variance extracted (AVE) and composite reliability (CR) values for each latent variable met the standards, indicating that the scale possesses good convergent and discriminant validity.The questionnaire design strictly adhered to the 20 indicators across 4 dimensions established in the evaluation framework (Table 2). A systematic review of the literature was conducted to organize research findings in related fields, ensuring the scientific rigor and validity of the questionnaire.

**Table 2:** Measurement scale.

Latent variable	Coding	Item	References
Technical Function Experience (TE)	TE1 Function Adaptation	The cultural and creative products generated by AIGC demonstrate high accuracy in analyzing the characteristics of cultural relics.	[18] [19] [20]
	TE2 Requirements are met	The generated results are highly consistent with the design requirements I entered	
	TE3 Material suitability	The product’s physical properties, such as size and material, are suitable for its intended use	
	TE4 It has a clear interface	The generated interface has a clear functional layout, and the required tools and options are easy to find	
	TE5 It is stable and runs smoothly	When an operational error occurs, the system provides clear solutions or guidance	
Cultural Aesthetic Value (CV)	CV1 Color Matching	The overall color scheme is artistic and appealing, in harmony with the museum’s cultural atmosphere.	[23] [24] [25] [26]
	CV2 pattern	The shape or pattern design is unique and features memorable visual elements	
	CV3 It offers outstanding advantages	The design showcases the unique strengths of AIGC technology (e.g., complex pattern generation)	
	CV4 Aesthetic Layering	The superimposed effect of a multidimensional aesthetic experience is prominent (such as the composite perception of visual patterns, tactile textures, and cultural narratives)	
Psychological Perception (PP)	PP1 Rich and diverse content	I believe AIGC technology can enhance the diversity of cultural and creative design	[11] [35] [36] [37]
	PP2 Cultural Association	This product reminds me of relevant historical stories or cultural scenes	
	PP3 Expressing preferences	I can express my personal preferences (e.g., style, elements) during the design process	
	PP4 Personal Fit	When I see the product, I feel a sense of identity or connection to traditional culture	
	PP5 Intelligent Trust	When using AIGC to generate cultural and creative products, you don’t have to worry about operational errors or poor results.	
User Acceptance (UA)	UA1 Social Identity	The cultural identity of the group that the product can embody	[44] [45] [46] [47]
	UA2 Recommended Products	Overall, I am satisfied with this cultural and creative product	
	UA3 Purchase physical items	I will recommend museum cultural and creative products generated by AIGC to others	
	UA4 Purchase intention	I might buy a physical cultural product featuring this design	
	UA5 Value Match	In the future, I will consider purchasing other museum cultural and creative products generated by AIGC again.	
	UA6 Value Recognition	The price of this cultural and creative product aligns with its cultural and practical value.	

### 3.3 Data collection and analysis

The sample data for this study primarily comes from the 18–45 age group. According to the Global Museum Cultural and Creative Consumption Report (2024) [56], the 18–45 age group accounts for 78% of total museum cultural and creative product consumption. They are the core consumer force in the current market, and as early adopters of AIGC technology, they possess greater awareness and experience with AIGC-generated products, enabling them to more accurately assess the integration of technology and culture.Data collection took place from January 15 to March 30, 2025, using a stratified random sampling design conducted online. Stratification variables included gender, age (18–25, 26–35, 36–45), and education level (high school or below, bachelor’s degree, master’s degree or above) to ensure the sample structure aligned with the demographic characteristics of the core consumer group and enhance representativeness.During the sampling process, the sample size for each stratum was first determined based on the population distribution ratio of each stratification variable, and then distributed via a targeted online survey platform (Questionnaire Star) to avoid sampling bias. To ensure data quality, two screening questions were included in the questionnaire design to verify the respondent’s age range and their experience with the design and consumption of museum cultural and creative products.During the data analysis phase, ineligible questionnaires were excluded, retaining only valid responses. The specific survey process is as follows: 1. Pre-launch phase (January 15–February 28): 80 pre-survey questionnaires were distributed, yielding 76 valid responses, which were used to revise the questionnaire; 2. Main Survey Phase (March 1–March 30): 350 formal questionnaires were distributed. To prevent invalid responses, features such as IP deduplication, response time limits, and logic validation provided by Questionnaire Star were utilized; 3. After questionnaire collection, invalid responses were manually screened, resulting in 323 valid responses, with a response rate of 92%.

Demographic analysis (Table 3) shows that the gender ratio of respondents was approximately 4:5, which is generally consistent with the gender characteristics of the primary consumer group for museum cultural and creative products.In the survey on the application of AI-generated art design (using a 5-point rating scale), the average score was 3.52, indicating that most respondents have some awareness of this field; the interest score reached 4.5, fully demonstrating consumers’ strong interest in this emerging art form, which suggests its strong market appeal and development potential.

**Table 3:** Participant characteristics (n = 323).

Measurement	Item	Frequency	Percentage
Gender	Male	146	45.2%
	Female	177	54.8%
Age	18–25	134	45.5%
	26–35	94	29.1%
	35–45	95	25.9%
Educational Attainment	High school or below	39	12.7%
	Junior college	56	17.34%
	Bachelor’s degree	150	46.44%
	Master’s degree or higher	78	24.15%
Frequency of purchasing museum cultural and creative products (per year)	1–3	107	33.13%
	4–6	91	28.17%
	6–10	96	29.72%
	>10	29	8.98%
Level of Understanding of Museum Culture	4.02		
Cognitive Level of AI Art Design	3.58		
Level of Interest in AI Art Design	4.02		

This study is a non-retrospective study conducted via a questionnaire survey; the research process did not involve human experimentation. All participants were voluntary respondents to the social survey, and data collection was strictly anonymized.Since all necessary data were collected during the aforementioned recruitment period, the authors were unable to access any personally identifiable information of the participants either during or after data collection. All participants signed written informed consent forms, which were archived in the form of photographic records and have received exemption approval from the Tsinghua University Ethics Review Committee (Approval No.: THU-04-2026-0074).The research process strictly adhered to the ethical principles of the Declaration of Helsinki. All participants signed written informed consent forms (archived electronically), and data collection was anonymized to ensure the privacy and security of participants.”

## 4 Results

### 4.1 Reliability analysis

This study utilized SPSS 26.0 software and the widely recognized Cronbach’s α coefficient to assess scale reliability. Cronbach’s α is a core indicator for measuring internal consistency reliability, reflecting the extent to which all items on a scale measure the same latent variable; its value ranges from 0 to 1.Most scholars argue that a coefficient value exceeding 0.9 indicates excellent scale reliability; a value between 0.8 and 0.9 indicates good reliability; a value between 0.7 and 0.8 is within an acceptable range; 0.6–0.7 indicates moderate reliability; 0.5–0.6 indicates low reliability; and if the value is below 0.5, the questionnaire design typically needs to be re-optimized.Test results show that the Cronbach’s α coefficient for the questionnaire in this study is 0.934, indicating a high level of reliability and strong data reliability (Table 4).

**Table 4:** Reliability and validity analysis.

Reliability Analysis			
Cronbach’s Alpha Coefficient	Standardized Cronbach’s α	Number of Items	Sample Size
0.934	0.934	20	323
**Validity Analysis (KMO Test and Bartlett’s Test)**			
KMO Value			0.955
Bartlett’s Sphericity Test	Approximate Chi-Square		3199.341
	DF		190
	*P*		0.000***

Significance levels: *** *p* < 0.001, ** *p* < 0.01, * *p* < 0.05.

### 4.2 Effect analysis

The KMO value was 0.955, far exceeding the threshold of 0.7, indicating strong correlations among the variables and that the data are highly suitable for factor analysis;The approximate chi-square value for Bartlett’s sphericity test was 3,199.341 (degrees of freedom df = 300), with a corresponding p-value of 0.000, which is significant at the 1% level. This rejects the null hypothesis that the variables are independent of one another, further validating the significant correlation among variables. This satisfies the prerequisites for factor analysis, indicating that the data possess extremely high construct validity and are suitable for extracting common factors through factor analysis (Table 4).

### 4.3 Conventional method bias (CMB) test

Since the data in this study were collected via a one-time self-report questionnaire, common method bias (CMB) may be present. We addressed this issue through procedural controls during the study design phase and statistical tests during the data analysis phase.

During data collection, we implemented the following measures to mitigate CMB risks at the source: (1) assured all participants that the questionnaire was completely anonymous and that data would be used solely for academic research, to reduce social desirability bias; (2) randomized the order of items measuring different constructs to prevent participants from guessing the hypothesized relationships between variables; (3) included reverse-scored items in the scales.Statistical Tests: After data collection, we conducted two statistical tests.

#### 4.3.1 Harman’s single-factor test.

An unrotated exploratory factor analysis was performed on all measurement items (Table 5). The results showed that there were multiple factors with eigenvalues greater than 1. The first factor explained 44.83% of the total variance (see Table 5). Although this value exceeds 40%, existing research by Polas et al. have confirmed that a value below the commonly used standard of 50% indicates the absence of a single, absolutely dominant common factor, suggesting a low risk of CMB [57].

**Table 5:** Summary of analysis of total variance explained, pearson correlation and AVE root, average variance extracted (AVE) value, model fit statistics, factor loadings and item discrimination.

Total Variance Explained										
Component	Variance Explained Before Rotation				Variance Explained After Rotation					
	Eigenvalue	Percentage of variance explained (%)	Cumulative Variance Explained (%)		Eigenvalue	Percentage of variance explained		Cumulative percentage of variance explained (%)		
1	8.967	44.834	44.834		8.967	44.834		44.834		
2	1.304	6.518	51.352							
3	1.15	5.749	57.101							
4	0.901	4.503	61.604							
5	0.747	3.734	65.338							
6	0.704	3.52	68.857							
7	0.637	3.185	72.042							
8	0.615	3.075	75.118							
9	0.599	2.995	78.113							
10	0.548	2.74	80.853							
11	0.52	2.6	83.454							
12	0.488	2.441	85.894							
13	0.467	2.335	88.229							
14	0.456	2.28	90.509							
15	0.392	1.962	92.471							
16	0.376	1.88	94.351							
17	0.349	1.743	96.094							
18	0.299	1.495	97.588							
19	0.286	1.429	99.018							
20	0.196	0.982	100							
Pearson correlation and AVE root, Average Variance Extracted (AVE) value										
		TE	CV			PP	Average Variance Extracted (AVE)	Composite Reliability (CR)		
TE		0.683					0.467	0.813		
CV		0.568 (0.000***)	0.696				0.485	0.788		
PP		0.687 (0.000***)	0.621 (0.000***)			0.712	0.502	0.833		
UA		0.644 (0.000***)	0.57 (0.000***)			0.721 (0.000***)	0.580	0.890		
Model fit statistics										
Common Measures	χ²	df	P	Chi-Square-to-Degrees-of-Freedom Ratio	GFI	RMSEA	RMR	CFI	NFI	NNFI
Criteria	–	–	>0.05	<3	>0.9	<0.10	<0.05	>0.9	>0.9	>0.9
Value	161.956	164	0.53	0.988	0.951	0	0.022	1.001	0.951	1.001
**Factor Loadings and Item Discrimination Analysis**										
Factor	Variable	Non-standard loadings	Standardized loading coefficients	z	SE	T	P	Group (mean ± standard deviation)		
TE	TE 1	1	0.604	–	–	−36.235	–	1.125 ± 0.333	2.007 ± 0.082	3.261 ± 0.442
	TE 2	1.031	0.608	8.835	0.117	−30.718	0.000***	1.239 ± 0.429	2.143 ± 0.351	3.307 ± 0.464
	TE 3	1.15	0.694	9.72	0.118	−24.882	0.000***	1.42 ± 0.496	2.245 ± 0.431	3.318 ± 0.515
	TE 4	1.235	0.735	10.098	0.122	−22.84	0.000***	1.636 ± 0.484	2.354 ± 0.48	3.489 ± 0.587
	TE 5	1.27	0.761	10.324	0.123	−22.717	0.000***	1.727 ± 0.448	2.653 ± 0.478	3.58 ± 0.62
CV	CV 1	1	0.608	–	–	−22.793	–	1.0 ± 0.0	1.503 ± 0.502	2.557 ± 0.641
	CV 2	0.977	0.605	8.547	0.114	−26.948	0.000***	1.0 ± 0.0	1.68 ± 0.468	2.727 ± 0.601
	CV3	1.222	0.734	10.227	0.119	−27.847	0.000***	1.0 ± 0.0	1.748 ± 0.435	2.727 ± 0.582
	CV4	1.268	0.734	10.231	0.124	−31.986	0.000***	1.0 ± 0.0	1.878 ± 0.329	2.909 ± 0.56
PP	PP 1	1	0.627	–	–	−48.407	–	1.0 ± 0.0	1.871 ± 0.337	3.193 ± 0.425
	PP 2	1.03	0.608	9.349	0.11	−42.211	0.000***	1.0 ± 0.0	2.075 ± 0.372	3.318 ± 0.515
	PP 3	1.135	0.691	10.357	0.11	−26.066	0.000***	1.284 ± 0.454	2.265 ± 0.443	3.341 ± 0.585
	PP 4	1.327	0.761	11.124	0.119	−26.296	0.000***	1.307 ± 0.464	2.381 ± 0.487	3.489 ± 0.625
	PP 5	1.426	0.832	11.829	0.121	−24.901	0.000***	1.557 ± 0.5	2.605 ± 0.49	3.67 ± 0.62
UA	UA 1	1	0.613	–	–	−31.689	–	1.0 ± 0.0	1.85 ± 0.358	3.261 ± 0.669
	UA 2	1.077	0.647	9.859	0.109	−42.35	0.000***	1.0 ± 0.0	2.129 ± 0.552	3.307 ± 0.511
	UA 3	1.216	0.703	10.505	0.116	−27.679	0.000***	1.239 ± 0.429	2.395 ± 0.49	3.557 ± 0.658
	UA 4	1.405	0.8	11.522	0.122	−25.56	0.000***	1.398 ± 0.492	2.497 ± 0.502	3.716 ± 0.694
	UA 5	1.549	0.846	11.961	0.13	−33.022	–	1.545 ± 0.501	2.694 ± 0.462	4.102 ± 0.526
	UA 6	1.675	0.9	12.426	0.135	−37.586	0.000***	1.614 ± 0.49	2.83 ± 0.444	4.273 ± 0.448

Significance levels: *** *p* < 0.001, ** *p* < 0.01, * *p* < 0.05.

Variable Full Names: TE: Technical Function Experience; CV: Cultural Aesthetic Value; PP: Psychological Perception; UA: User- Acceptance.

#### 4.3.2 Confirmatory factor analysis.

Since all data in this study were collected via self-report questionnaires, confirmatory factor analysis was conducted to avoid common method bias (CMB) affecting the reliability of the results (Table 5). The results show that the four-factor model in this study exhibits excellent fit indices (χ²/df = 0.988,GFI = 0.951, RMSEA = 0.000, RMR = 0.022, CFI = 1.001, NNFI = 1.001) (Table 5); simultaneously, the square roots of the AVE for each latent variable were greater than the intercorrelation coefficients between factors, and the intercorrelation coefficients ranged from 0.678 to 0.832, with no extremely high correlations (Table 5).The average variance extracted (AVE) values for TE and CV were 0.467 and 0.485, respectively. Although slightly below the strict threshold of 0.5, both met the general lenient standard of ≥0.4 commonly used in social science research, and the composite reliability (CR) was well above the passing threshold of 0.7.According to the classic criteria of Fornell & Larcker (1981) [58], when CR ≥ 0.7, even if AVE is slightly below 0.5, convergent validity can still be considered acceptable; combined with the large sample size (N = 323) in this study, the data robustness is good, and does not affect the validity of the model or the reliability of the conclusions.Taken together, these findings indicate that this study does not exhibit serious common-method bias, and the data quality is reliable.

### 4.4 Item discrimination analysis

This subsection verifies the rationality of the scale item design through item discrimination analysis. Specific analytical indicators include mean ± standard deviation, t-test results, and significance P-values. The analytical logic is as follows: if the P-value for each item is significant (P < 0.05), it indicates that the item can effectively distinguish between respondents at different levels, and the item design is reasonable;if the P-value is not significant, it indicates that the item cannot effectively distinguish between levels and should be deleted. The results show that the P-values for all items in Table 5 are 0.000*** (P < 0.001), significantly rejecting the null hypothesis, indicating that the items in the scale possess good item discrimination and are reasonably designed.

### 4.5 Structural equation modeling analysis

**Fig 2:**
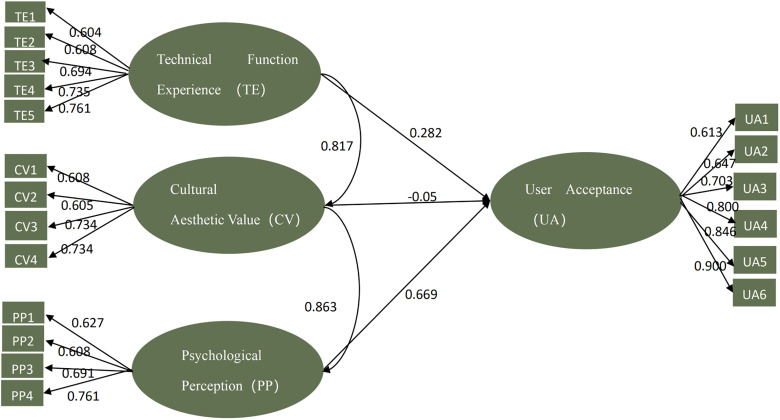
Results of structural equation modeling analysis. The model presents the empirical paths, standardized path coefficients, and factor loadings for the proposed conceptual framework. (1) Measurement Model: The rectangles (TE1–TE5, CV1–CV4, PP1–PP4, and UA1–UA6） represent the observed indicators, with the numerical values on the arrows indicating their respective factor loadings onto the four latent variables. (2) Structural Model: The single-headed arrows indicate the standardized path coefficients from the independent latent variables to User Acceptance (UA). Specifically, Technical Function Experience (TE) (β = 0.282) and Psychological Perception (PP) (β = 0.669) exhibit positive effects on UA. Conversely, Cultural Aesthetic Value (CV) shows a negligible negative path coefficient to UA (β = −0.05). (3) Correlations: The double-headed curved arrows represent the correlation coefficients among the exogenous latent variables, showing strong positive correlations between TE and CV (*γ* = 0.817), as well as between CV and PP (*γ* = 0.863).

To investigate the influence of TE, CV, and PP on UA, a structural equation model of latent variables was established. The mediating effects of CV and PP were also analyzed, with the results shown below (Fig 2).

#### 4.5.1 Factor loadings analysis.

A factor loading table typically includes latent variables, analysis items, unstandardized loadings, and z-test res\ults. Factor loadings are used to screen measurement variables within a factor.Generally, if a measurement variable passes the significance test (P < 0.05) and its standardized loading coefficient is greater than 0.5, it indicates that the measurement variable meets the factor requirements. If the conditions differ significantly, consider removing the variable. If the measurement relationship is good, the standardized loading coefficient is typically greater than 0.5. (Table 6)

**Table 6:** Results of model fit, regression coefficients, and mediation effect tests.

Model Fit Analysis											
Common Metrics	X²	df	P	χ²/df	GFI	RMSEA	RMR	SRMR	CFI	NFI	NNFI
Criteria	–	–	>0.05	<3	>0.9	<0.10	<0.05	<0.1	>0.9	>0.9	>0.9
Value	232.434	166	0.001	1.400	0.928	0.035	0.031	0.040	0.979	0.929	0.975
**Model Regression Analysis**											
**Factor (Latent Variable)**	**—**	**Analytical Variable (Observed Variable)**		**Unstandardized Coefficients**	**Standardized Coefficients**	**Standard error**		**Z**		***P***	
TE	—	CV		0.735	0.817	0.091		8.096		0.000***	
CV	—	PP		1.031	0.863	0.12		8.605		0.000***	
TE	—	UA		0.319	0.282	0.116		2.752		0.006***	
CV	—	UA		−0.074	−0.059	0.214		−0.344		0.731	
PP	—	UA		0.704	0.669	0.149		4.716		0.000***	
**Mediating Effect Test Results (SEM)**											
**Effect**	**Term**			**Effect Size**	**Standard Error**	**Lower Limit of 95% Confidence Interval**		**Upper limit of 95% confidence interval**		**Significance**	
Direct effect	FE => UA			0.144	0.062	0.021		0.266		Significant	
	CV => UA			0.163	0.065	0.035		0.292		Significant	
Indirect effect	FE => PP => PP => UA			0.015	0.007	0.103		0.343		Significant	
	CV => PP => PP => UA			0.01	0.005	0.177		0.405		Significant	
Total effect	FE => UA			0.291	0.06	0.173		0.409		Significant	
	CV => UA			0.253	0.067	0.121		0.384		Significant	

Significance levels: *** *p* < 0.001, ** *p* < 0.01, * *p* < 0.05.

Variable Full Names: TE: Technical Function Experience; CV: Cultural Aesthetic Value; PP: Psychological Perception; UA: User- -Acceptance.

The results of the factor loading table show that the standardized loadings of all measurement variables on their corresponding latent variables are highly statistically significant (p < 0.001), indicating that the measurement model possesses ideal validity.In the FE analysis, the standardized loadings for each item ranged from 0.604 to 0.761, CV ranged from 0.605 to 0.734, PP ranged from 0.627 to 0.832, and UA ranged from 0.613 to 0.900, all exceeding the threshold of 0.5, reflecting that each observed variable effectively explains the variance of the latent variable.It is particularly noteworthy that the loadings for UA exhibit a clear increasing trend (UA1–UA6: 0.613–0.900), indicating that higher-order items possess stronger representational capacity for the latent variable.The z-values corresponding to all unstandardized coefficients are greater than the critical value of 7.88 (corresponding to p < 0.001), and the standard errors are within the range of 0.109–0.135, indicating that the parameter estimates have excellent precision.The loading coefficient of PP6 in the PP scale (0.832) and that of UA6 in the UA scale (0.900) are close to the upper limit of 0.9, suggesting a possible slight redundancy, but this remains within an acceptable range. Overall, this measurement model meets the requirements for convergent validity, and each item effectively reflects the theoretical construct of its corresponding latent variable.

#### 4.5.2 Model fit analysis.

Model fit indices include the chi-square-to-degrees-of-freedom ratio, GFI, RMSEA, RMR, SRMR, CFI, NFI, and NNFI. The chi-square value and degrees of freedom are primarily used to compare multiple models. A smaller chi-square value is preferable, while degrees of freedom reflect the model’s complexity—the simpler the model, the more degrees of freedom it has; conversely, the more complex the model, the fewer degrees of freedom it has. GFI (Goodness-of-Fit Index): This primarily assesses the model’s fit to the sample observations through the coefficient of determination and regression standard error. Its range is 0–1; values closer to 0 indicate poorer fit. When GFI ≥ 0.9, the model is considered to fit well. RMSEA (Root Mean Square Error of Approximation): Typically, RMSEA should be below 0.08(smaller values are better). RMR (Root Mean Square Residual): This metric measures model fit by calculating the average residual between predicted and actual observed correlations. If RMR < 0.1, the model is considered to fit well.CFI (Comparative Fit Index): In a comparison between the hypothesized model and an independent model, this index ranges from 0 to 1. A value closer to 0 indicates poorer fit, while a value closer to 1 indicates better fit. Generally, when CFI ≥ 0.9, the model is considered to fit well. NNFI (Non-normalized Fit Index) and CFI (Comparative Fit Index): A higher value indicates better performance of the fitted model.

The fit indices for this structural model are shown in Table 5: χ²/df = 1.400, RMSEA = 0.035, CFI = 0.979, SRMR = 0.040.These metrics all meet the recommended standards (χ²/df < 3, RMSEA < 0.1, CFI > 0.90, SRMR < 0.1), indicating a good fit between the model and the data.

#### 4.5.3 Model regression analysis.

Table 6 presents the regression coefficients for the path nodes, which can be interpreted as a simple linear regression using the least squares method. Typically, examining the p-value and the standardized path coefficient is sufficient to determine whether there is a direct linear effect along the path (X—Y).Based on the significance test analysis (P < 0.05), we can determine whether an influence relationship exists between the model variables. If a significant difference is found, it indicates that an influence relationship exists between the variables; in this case, the standardized path coefficients can be used to conduct an in-depth analysis of the efficiency of the influence.

The regression coefficient table for the structural equation model shows that, with the exception of the CV—UA path *P* = 0.619, which did not pass the significance test, all path coefficients are statistically significant at the 1% significance level (P < 0.01).Specifically, the model’s path coefficient table reveals the following: For the paired factor TE—CV, the significance P-value is 0.000***, reaching the significance level; therefore, this path is valid, with an effect coefficient of 0.817.For the paired factor CV—PP, the significance P-value is 0.000***, reaching the significance level; therefore, this path is valid, with an effect coefficient of 0.863. For the paired factor PP—UA, the significance P-value is 0.000***, reaching the significance level; therefore, this path is valid, with an effect coefficient of 0.669.For the paired item TE—UA, the significance P-value is 0.004***, reaching the significance level; therefore, this path is valid, with an effect size of 0.282. For the paired factor CV—UA, the significance P-value is 0.619, failing to reach the significance level; therefore, this path is invalid.

#### 4.5.4 Results of the mediation effect test.

This study employed Structural Equation Modeling (SEM) to test the chained mediation model, with Technical Function Experience (TE) as the independent variable, Cultural Aesthetic Value (CV) as the antecedent variable, Psychological Perception (PP) as the chained mediator, and User-Acceptance (UA) as the dependent variable, constructing the following path model: FE – CV – PP – UA

The model employed 5,000 Bootstrap samples, and the significance of the mediating effect was assessed using a 95% confidence interval (CI); if the interval did not include 0, the effect was considered significant.The overall structural equation model fits well, with all indices meeting ideal standards: χ²/df = 0.988, GFI = 0.951, RMSEA = 0.000, RMR = 0.022, CFI = 1.001, and NNFI = 1.001.

Bootstrap test results show that both chain mediation paths are significant, as shown in Table 6. The results indicate that Psychological Perception (PP) plays a significant chain mediating role between Technical Function Experience (TE) and User- -Acceptance (UA), as well as between Cultural Aesthetic Value (CV) and User- -Acceptance (UA).That is, FE and CV can positively influence user acceptance by enhancing psychological perception, and the research hypothesis is supported.

## 5 Discussion

Based on an integrated framework combining the Technology Acceptance Model (TAM), the Value Acceptance Model (VAM), and the Stimulus-Organism-Response (S-O-R) model, this study used Structural Equation Modeling (SEM) to validate the user acceptance mechanism of AIGC-generated museum cultural and creative products. The aim was to deeply analyze the intrinsic relationships among technical functional experience, cultural aesthetic value, psychological perception, and user acceptance, while addressing controversies and gaps in existing research.Key findings indicate that Technical Functional Experience (TE) has a significant direct positive effect on User Acceptance (UA), while also indirectly influencing UA through the chain mediation pathway of “Cultural Aesthetic Value (CV) — Perceived Perception (PP).” CV has a significant positive effect on PP, but its direct effect on UA is not significant; PP has a significant positive effect on UA.In terms of measurement model quality, the composite reliability (CR) of all latent variables exceeded 0.7. The average variance extracted (AVE) met the standard for PP and UA, while those for TE and CV were slightly below 0.5. Furthermore, the square roots of the AVE for all latent variables were greater than the intercorrelation coefficients among factors, indicating that the scales possess good convergent and discriminant validity;The fit indices for both the four-factor confirmatory factor analysis model and the chained mediation SEM model met ideal standards. The common method bias test did not reveal any severe common-source bias, and the data results are reliable, laying a solid foundation for subsequent theoretical interpretation and dialogue with the literature.

In integrating and engaging with existing literature, this study first confirms the strong positive influence of TE on CV, which aligns with the view proposed by Han S et al. (2022) that “technological precision is a prerequisite for the effective translation of cultural symbols” [31].The technical experience provided by AIGC algorithms—including the accuracy of analyzing cultural relic features and operational fluidity—directly determines the authenticity of cultural symbol transformation and the integrity of aesthetic expression. This finding addresses the limitations of the Technology Acceptance Model (TAM) in cultural contexts—— traditional TAM focuses on the attributes of technological tools, whereas this study confirms that technological functions must be transformed through cultural values to maximize their driving effect on user acceptance. This resonates with the research conclusion by Zhang LY et al. (2026) that “cultural context variables are key to the cross-scenario adaptability of the Technology Acceptance Model” [11], providing further theoretical support for research on the “technological attributes — cultural cognition” transmission mechanism. Second, the “TE—CV—PP—UA” chain-mediated pathway revealed in this study aligns closely with the core logic of the S-O-R model—“stimulus—organism—response” [9}.Among these, CV, as the critical link in the transformation from “technological stimulus” to “psychological organism,” exerts a strong driving effect on PP, corroborating Shi et al.’s assertion that “cultural value perception is the core mediator in the transformation of emotions and behavior” [22};Meanwhile, the significant influence of PP on UA supports the view proposed by Pang C et al. that “cultural identity is the direct antecedent of purchase intention for cultural and creative products” [23]. Unlike existing studies that often focus on a single mediating variable, this study confirms the chain-like transmission effect between cultural aesthetic value and psychological perception, thereby refining the theoretical chain of “technology–culture–psychology–behavior” and providing a more nuanced mechanistic explanation for user acceptance research in the field of cultural heritage digitization.

Given the lack of statistical significance in the CV—UA pathway, this study does not simply dismiss it as an “ineffective pathway,” but instead conducts an in-depth analysis by integrating cultural consumption theory:Cultural aesthetic value is essentially an objective perception of the quality of cultural symbol translation and the intensity of emotional resonance, whereas user acceptance is a complex decision-making process encompassing attitudes, intentions, and behaviors. It requires psychological internalization processes such as cultural identification and aesthetic pleasure to achieve the leap from “value recognition” to “behavioral inclination” [35]. This finding aligns with the observation by Tubadji et al. that “the mere reproduction of cultural symbols cannot directly drive consumption behavior” [28], indicating that the behavioral driving effect of cultural aesthetic value is context-dependent. Its impact manifests only through the mediation of psychological perception rather than acting directly on acceptance behavior.This non-significant result is not a flaw in the model but rather validates the complex transmission mechanisms of user acceptance behavior within a cultural context, avoiding simplistic linear assumptions about variable relationships. By providing a reasonable interpretation rather than dismissing the non-significant result, the study enhances its methodological rigor.

In defining theoretical contributions, this study consistently adheres to the boundaries of empirical support and avoids excessive extrapolation. The theoretical advancements of this research are primarily reflected in three aspects: First, it expands the cultural adaptability of the Technology Acceptance Model (TAM) by integrating CV and PP variables to construct a three-stage acceptance framework of “Technology Function – Cultural Transformation - Psychological Internalization” three-stage adoption framework, addressing the limitation of traditional TAM in cultural contexts that overlooks non-utilitarian variables; second, it refines the application of the S-O-R model to digital cultural products, clarifying the transmission pathways of “technology + culture” dual stimuli, and confirming the key roles of CV as a “stimulus converter” and PP as a “core organism”;Third, the study refined research on the mediating mechanisms of cultural value perception, revealing the chain-like transmission effects between CV and PP.At the same time, this study clearly defines its scope: the research model focuses on museum cultural and creative products generated by AIGC, and the generalizability of its conclusions requires further validation in other digital cultural product contexts; the study did not conduct cross-cultural comparisons, nonlinear tests, or moderation effect analyses, and the relevant discussions in the text are based solely on theoretical inferences from existing literature rather than empirical validation of conclusions;The AVE values for TE and CV in the model are slightly below 0.5. Although the CR values meet the criteria and the factor loadings are significant—consistent with Fornell & Larcker’s (1981) standard that “aggregate validity can still be considered acceptable when AVE is slightly low but CR meets the criteria” [58]—this still suggests room for optimization of the scales, which to some extent limits the robustness of the theoretical conclusions.

Based on the above research findings, this study offers targeted practical implications: In terms of technical optimization, given the high path coefficient of TE—CV, it is recommended that AIGC algorithm optimization focus on the accuracy of cultural symbol analysis. By expanding the museum artifact feature database, the algorithm’s ability to identify and reconstruct cultural elements can be enhanced, thereby reducing the error rate in symbol translation. Concurrently, operational workflows should be simplified to lower the technical barrier for users and strengthen the positive influence of the technological experience on cultural aesthetic value;Regarding cultural empowerment strategies, in light of the strong transmission effect of CV—PP,AIGC design for museum cultural and creative products should emphasize the profound expression of cultural connotations. It must not only accurately reproduce artifact symbols but also incorporate historical narratives and elements of emotional resonance—such as enhancing users’ cultural identity through dynamic presentations of the historical stories behind artifacts. Furthermore, color schemes and pattern designs should be optimized for cultural appropriateness to ensure visual expressions align with the museum’s cultural atmosphere, thereby improving the efficiency of converting cultural aesthetic value into psychological perception;Regarding psychological drivers, given the significant influence of the PP—UA pathway, psychological perception can be elevated by enhancing user engagement. This can be achieved by allowing users to express personal preferences during the design process to satisfy their need for autonomy, and by incorporating cultural interaction segments where users participate in the design of cultural symbol combinations, thereby strengthening their emotional connection to the product and ultimately fostering acceptance behavior. These practical insights are derived from empirical findings and are highly actionable.

This study still has certain limitations and could be further deepened in multiple aspects in the future: the sample structure is biased, with a higher proportion of young people aged 18–35, which differs from the age distribution of the actual consumer group for museum cultural and creative products;the insufficient representation of middle-aged and elderly individuals may limit the applicability of the conclusions to this demographic. Future research should optimize the sample structure by increasing the proportion of middle-aged and elderly participants and conducting comparative analyses across age groups. Standardized measurement of technology anxiety and cultural nostalgia among this demographic using established validity and reliability scales could provide reliable methodological support for testing moderating effects, thereby enhancing the external validity and generalizability of the findings;The study employed a cross-sectional design, capturing only user attitudes and intentions at a specific point in time, and was unable to track the dynamic changes in user acceptance mechanisms as technology evolves or consumption experiences accumulate. Future research could adopt a longitudinal tracking design to collect user data periodically and explore dynamic evolutionary processes; the model did not include moderating variables, such as user digital literacy, frequency of cultural participation, and product type, which may have overlooked the influence of these variables on the core pathways. Future research could introduce moderating variables to test their mechanisms of action;The study did not conduct cross-cultural comparative research, and the cross-cultural applicability of the conclusions remains to be verified. By comparing differences in acceptance mechanisms between Eastern and Western users, theoretical support for cross-cultural communication could be provided. Additionally, the scale design needs to be optimized by adding targeted items for the TE and CV dimensions to improve their AVE values and further enhance the quality of the measurement model.By objectively analyzing these limitations and clarifying future research directions, this study provides a clear path for subsequent exploration in this field

## 6 Conclusion

This study examines AIGC-generated museum cultural and creative products. Based on 323 valid questionnaire responses, Structural Equation Modeling (SEM) was employed to examine the relationships among Technical Functional Experience (TE), Cultural Aesthetic Value (CV), Psychological Perception (PP), and User Acceptance (UA), yielding the following core conclusions:Technical Functional Experience has a significant direct positive impact on user acceptance and also indirectly influences user acceptance through the chain-mediated pathway of “Cultural Aesthetic Value — Psychological Perception,” confirming the synergistic driving effect of technical functionality and cultural value;Cultural aesthetic value has a significant positive impact on psychological perception, but its direct impact on user acceptance is not significant, indicating that cultural aesthetic value must undergo a process of psychological internalization before it can be transformed into acceptance behavior; psychological perception has a significant positive impact on user acceptance and serves as the key link connecting cultural aesthetic value and acceptance behavior; the research scales demonstrate good reliability, validity, and discriminant validity, the model fit indices are excellent, and there is no severe common method bias, ensuring the reliability of the data results.It should be noted that this study employed a cross-sectional design, which captures only a snapshot of users’ attitudes and behavioral intentions toward AIGC-generated museum cultural and creative products at a specific point in time. It cannot track the dynamic changes in user acceptance mechanisms resulting from AIGC technology iterations or the accumulation of cultural consumption experiences.

The theoretical value of this study lies in integrating the TAM, VAM, and S-O-R models to construct a technology acceptance framework tailored to cultural contexts, revealing a three-stage transmission mechanism of “technological functionality—cultural transformation—psychological internalization”; Its practical significance lies in providing a three-dimensional pathway of “technological precision, cultural depth, and psychological interactivity” for the design and optimization of AIGC cultural and creative products, offering empirical references for the digital innovation of museum cultural and creative products. The study has limitations such as sample structural bias and a cross-sectional design; future research could be further deepened by optimizing sample structure, adopting a longitudinal design, and introducing moderating variables.Overall, this study reveals the user acceptance mechanism for AIGC-generated museum cultural and creative products, offering new perspectives and references for theoretical research and practical applications in the field of cultural heritage digitization.
